# Immunological Responses to Total Hip Arthroplasty

**DOI:** 10.3390/jfb8030033

**Published:** 2017-08-01

**Authors:** Kenny Man, Lin-Hua Jiang, Richard Foster, Xuebin B Yang

**Affiliations:** 1 Biomaterials and Tissue Engineering Group, School of Dentistry, University of Leeds, Leeds LS2 9JT, UK; mnkklm@leeds.ac.uk; 2 School of Biomedical Sciences, Faculty of Biological Sciences, University of Leeds, Leeds LS2 9JT, UK; l.h.jiang@leeds.ac.uk; 3 School of Chemistry, University of Leeds, Leeds LS2 9JT, UK; r.foster@leeds.ac.uk; 4 Medical College and the First Affiliated Hospital, Henan University of Science and Technology, Henan 471023, China

**Keywords:** total hip arthroplasty, implant materials, wear particles, immunological response

## Abstract

The use of total hip arthroplasties (THA) has been continuously rising to meet the demands of the increasingly ageing population. To date, this procedure has been highly successful in relieving pain and restoring the functionality of patients’ joints, and has significantly improved their quality of life. However, these implants are expected to eventually fail after 15–25 years in situ due to slow progressive inflammatory responses at the bone-implant interface. Such inflammatory responses are primarily mediated by immune cells such as macrophages, triggered by implant wear particles. As a result, aseptic loosening is the main cause for revision surgery over the mid and long-term and is responsible for more than 70% of hip revisions. In some patients with a metal-on-metal (MoM) implant, metallic implant wear particles can give rise to metal sensitivity. Therefore, engineering biomaterials, which are immunologically inert or support the healing process, require an in-depth understanding of the host inflammatory and wound-healing response to implanted materials. This review discusses the immunological response initiated by biomaterials extensively used in THA, ultra-high-molecular-weight polyethylene (UHMWPE), cobalt chromium (CoCr), and alumina ceramics. The biological responses of these biomaterials in bulk and particulate forms are also discussed. In conclusion, the immunological responses to bulk and particulate biomaterials vary greatly depending on the implant material types, the size of particulate and its volume, and where the response to bulk forms of differing biomaterials are relatively acute and similar, while wear particles can initiate a variety of responses such as osteolysis, metal sensitivity, and so on.

## 1. Introduction

Total hip arthroplasties (THA) have been incredibly successful in relieving pain from the suffered joints and restoring normal joint function, resulting in significant improvement in a patient’s quality of life. However, the implant may fail later on and require revision surgery, which can incur an increased risk of complications and additional costs. In 1989, Kavanagh et al. (1989) reviewed the performance of 166 Charnley THA performed at the Mayo Clinic after a minimum of 15 years postoperatively and reported that the probability of failure (revision or symptomatic loosening) was 0.9% at one year, 4.1% at five years, 8.9% at ten years, and 12.7% at fifteen years [1]. According to the UK National Joint Registry, the current revision rate benchmark at 10 years should be less than 5% [2]. In the US alone, over 40,000 THAs had to be revised in 2005 due to implant loosening, and it is expected that the rate of revision will increase by 137% by 2030 [3]. Aseptic loosening is the main cause for 40 revision surgery over the mid and long-term, and is responsible for more than 70% of hip revisions (this is usually accompanied by osteolysis, followed by infection and implant instability [4,5]). For metal-on-metal (MoM) THA, revisions can also be caused by fracture (7.69%), metal reactions (7.69%), infections (12.08%), instability (9.13%), manufacturer defect (6.73%), and miscellaneous (7.69%) [6]. Periprosthetic osteolysis is the term referring to the continuous resorption of bone in contact with the THA, which often predates asceptic loosening and is likely to be asymptomatic for many years [7]. As a result of the formation of these severe bone defects, patients often require revision surgeries. However, the revision surgeries are complicated procedures, which take longer to perform and are associated with higher costs and increased risk of further complications [7]. Incidence rates of osteolysis range between 5 and 20%, however this is expected to rise significantly over time due to the fact that approximately 40% of THA patients are below 65 years of age [8,9]. The survival of the prosthesis is affected by numerous factors including gender, age, type of prosthesis, pathology, and the skill of the surgeon. Bordini et al. (2007) performed a multivariate survival analysis on the aseptic loosening of 4750 THA between 1995 and 2000, and found that the worst conditions occurred in male patients who were below 40 years in age, afflicted with sequelae of congenital diseases, and in whom the THA procedure was performed by a surgeon who was less skilled (e.g., had performed less than 400 THA during the period assessed) [10]. The type of prosthesis also had an affect where uncemented cups and stems had a higher success rate compared to the less expensive cemented counterparts. With the increasing ageing population, the number of primary joint replacements is anticipated to increase in the future. Therefore, the ability to reduce the number of necessary revision surgeries could have a significant impact on the healthcare system [11].

One of the aims of using the prosthesis in THA is to replicate the natural joint function by mimicking the functional aspects of hyaline cartilage to allow for a durable, smooth, sliding interface, which provides stable and controlled movement of the joint [12]. These prostheses acquire a delicate balance between lubrication, friction, and wear. To achieve these, different materials such as ultra-high-molecular-weight polyethylene (UHMWPE), cobalt chromium (CoCr), and alumina ceramics are used extensively in THA [13,14]. However, these prosthetic materials can be lost, which is known as wear, due to excessive abrasion, fatigue, and adhesion at the articulating interfaces of the prosthesis. The wear debris and/or particles from these implant materials often activate numerous immunological reactions, which ultimately results in implant failure [15]. Therefore, this review mainly focuses on the immunological responses to the bulk implants and wear particles.

## 2. Biological Responses to Bulk Implant Materials

The normal host response to all types of implanted biomaterials, which is also known as the foreign-body response, becomes aberrant and prolonged. This response is initiated when the biomaterial is implanted and often causes local tissue damage where proteins, fluid, and blood cells escape from the vascular system via the exudation procedure, leading to the formation of an avascular, collagenous membrane at the tissue-material interface, which isolates the foreign material from the surrounding tissue [16]. During this procedure, proteins such as fibrinogen, the von Willebrand factor, and IgG are instantaneously adsorbed onto the implant surface. The interactions at the blood-material interface activates a complex series of events including protein adsorption, platelet, and leukocyte activation/adhesion, and the activation of the complement and coagulation systems, as well as inflammation [17,18]. Platelets are the first cell fragments to interact with the implant [19] and are activated by adsorbed proteins, leading to the release of bioactive molecules including platelet-derived growth factor (PDGF), adenosine diphosphate (ADP), serotonin, and histamines. Subsequently, histamines and neuropeptides increase vascular permeability, allowing plasma proteins to enter the tissue, which in turn activates the complement and kinin system, resulting in the attraction of polymorphonuclear leukocytes (PMN) such as neutrophils [20]. The expression of P-selectin on the endothelial cell membrane aids platelet adhesion to neutrophils, monocytes, and leukocytes. Furthermore, platelet activation initiates changes in its cellular morphology, which allows the expression of coagulation factors prothrombinase and tenase [18]. The formation of the fibrin clots at the implant surface is the provisional matrix, which contains a plethora of signalling molecules such as cytokines, mitogens, growth factors, and chemoattractants [16].

Inflammation is the body’s normal protective response to any injury (including surgery) or foreign bodies. Acute inflammation usually follows the early stage (protein adsorption) of the foreign-body response, as previously described [20,21]. Chemotactic agents within the provisional matrix play a key role in controlling the emigration of neutrophils from the vasculature. The travelling leukocytes surrounding the implant become activated in response to the cytokines released by the platelets, e.g., PDGF and β-thromboglublin [22]. Following localisation and activation of macrophages and neutrophils to the site of injury, enzymes are released, and then the neutrophils mediated phagocytosis occurs. Theoretically, this phagocytosis should include the procedures of firstly recognising and attaching to the foreign materials, then engulfing and degrading them. However, due to the materials size, engulfment and degradation are often not possible, although the process of recognition and attachment occurs. Instead, the implant materials are coated with opsonins such as complement activated fragments C3b and IgG, which aid the adhesion and activation of neutrophils and macrophages [21]. Macrophages assemble at the implant site, leading to further production of chemoattractive-signalling molecules such as PDGF, tumour necrosis factor (TNF-α), Interleukin 6 (IL-6), granulocyte-colony stimulating factor (G-CSF), and granulocyte macrophage colony stimulating factor (GM-CSF), leading to further recruitment of macrophages to the implant site [23].

At the end-stage of the foreign-body response, or when the chronic inflammation occurs, mononuclear cells such as monocytes, lymphocytes, and/or macrophages can present at the implant site. These macrophages, which are aided by the production of IL-4, IL-13 from Th2 lymphocytes, can fuse together to form a multinucleated foreign body giant cell (FBGC) at the implant surface [24,25]. Also, they can secrete different factors, which attract the activated fibroblast to produce excessive amounts of extracellular collagen matrix.

Next, the infiltrated fibroblast, macrophages, and neovasculization will present within the newly formed tissue (granulation tissue), which is a precursor for forming a fibrous capsule [16]. This capsule may continue to grow following inflammation due to mechanical motions or chemical leaching exerted in the joint. It was thought that the host response to most of bulk biomaterials used in THA follow these main stages; however, the response to the wear particles released by different biomaterials over time differs greatly [20,26].

## 3. Biological Responses to Wear Particles

### 3.1. Ultra-High Molecular Weight Polyethylene Wear Particles

UHMWPE still remain as the gold standard biomaterial for use in hip and knee replacements due to their high fracture toughness, superior wear resistance, and relative biocompatibility compared to other materials [27]. UHMWPE is made up of extremely long chains of polyethylene and each individual molecule adds strength to the whole structure through its length. In vitro, it has been shown that gamma radiation-crosslinked UHMWPE can decrease the wear reduction rate of the biomaterials by up to 96% compared to that without a crosslink [28]. However, recent studies have characterised the immunological response to the wear particles released during long-term implant use, resulting in periprosthetic osteolysis and implant loosening [29]. UHMWPE microparticles can cause an inflammatory cascade upon phagocytosis by macrophages and lead to osteolysis [30].

A number of studies have shown that the wear particles produced by the UHMWPE cup vary greatly in volume and size. These variations have been reported to elicit differing extents of the biological response. For example, a large number of macrophages are identified in the interfacial membrane of patients, and these cells are actively phagocytosing the wear particles [31]. It was suggested that larger particles (>10 μm) can be engulfed by giant cells, while smaller particles would be phagocytosed by macrophages [32].

In response to implant debris, the UHMWPE wear particles can activate macrophages residing within the tissue to produce primarily proinflammatory mediators, which can affect the local microenvironment surrounding the implants [33]. The activated macrophages release cytokines including interleukin-1 (IL-1), IL-3, IL-6, TNF-α, GM-CSF, macrophage-colony stimulating factor (M-CSF), stem cell factor (SCF), PDGF, matrix metalloproteinases (MMPs), and prostaglandin E_2_ (PGE_2_) [20,34]. These cytokines can act on osteoclast precursors (OCPs) and play a significant role in osteoclast development and activation [35,36].

The expression of chemokines by activated macrophages, osteoblasts, and fibroblasts is essential to the innate immune response to biomaterial debris. Chemokines such as chemokine ligand 2 (CCL2) and stromal cell-derived factor 1 (SDF1), also known as C-X-C motif chemokine 12 (CXCL12), further attract monocyte and/or macrophages from blood to the periprosthetic synovial-like fibrous membrane and then to the bone-implant interface [16,37,38]. Fibroblasts are also involved in osteoclastogenesis and bone resorption, where they secrete a plethora of factors in response to the particles, such as MMP-1, monocyte chemotactic protein-1 (MCP-1), IL-1β, IL-6, IL-8, cyclooxygenase 1 (COX-1), COX-2, and transforming growth factor beta (TGFβ). These stimulated fibroblasts are able to directly affect osteoclastogenesis by expressing the receptor activator of nuclear factor kappa-B ligand (RANKL) [33,39].

At the bone surface, the RANKL-RANK-osteoprotegerin (OPG) axis plays a key role in osteoclastogenesis [36]. TNF-α supports the survival and differentiation of osteoclasts, whereas IL-1β supports the activation of osteoclast [35]. Interestingly, M-CSF secreted from osteoblastic cells in the bone marrow can bind to and activate its receptor (M-CSFR) on the OCPs to induce the expression of RANK on the surface of OCPs. RANKL-RANK interaction can promote osteoclastogenesis and the formation of multinucleated osteoclast [36]. Recently, it has been revealed by Barrow et al. (2011) that another co-stimulatory molecule called the osteoclast-associa ted receptor (OSCAR) plays an important role in osteoclast differentiation, maturation, and activity. As a result, the hyperactivity of osteoclasts around the bone-implant interface can promote the release of cathepsin K (Cath-K), which is the primary collagenase in osteoclasts, and hydrochloric acid, resulting in osteolytic bone resorption [7,40].

As previously described, the activated macrophages attempt to phagocytose and degrade these particles, fusing with intracellular vesicles containing proteolytic enzymes. However, these materials are biologically inert and undegradable; thus, macrophages release numerous cytokines and mediators in the surrounding tissue, which recruits additional cells to combat the UHMPWE particles. Therefore, when the debris particles are too large to engulf, a foreign body granulomatous reaction occurs, and macrophages fuse together to form multinucleated giant cells (FBGC). These FBGC will “wall off” these particles to shield and isolate them from the surrounding tissue [20,41].

### 3.2. Metal Wear Particles

Cobolt chromium (CoCr) is commonly utilised in MoM THA for the articulating surfaces of the joint replacements due to their low wear rates. However, the degradation of the biomaterials through wear and corrosion produces large amounts of nanometer sized particles. It has been reported that wear particles produced from THA utilising this kind of biomaterial are <50 nm in size, with a wear rate of 4.2 µm per year [42,43]. Owing to their small size and large number [42], nanoparticles have the risk to be disseminated systemically [44], with possible cytotoxic, genotoxic, and immunological consequences in both local and distant microenvironments such as the liver, spleen, lymph nodes, and bone marrow [45,46,47].

Normally, metal wear particles are taken up by pinocytosis and endocytosis, whereas large particles are internalised by macrophages via the lysosomal pathway [48,49]. Chromate ions or Cr (VI) are negatively charged and tetrahedral in shape, which is thought to allow their passage through nonspecific anion channels, leading to their accumulation in the cytoplasm [50,51]. Cr (VI) undergoes reduction to Cr (III) by a series of networks involving molecules such as ascorbate and/or enzymes (e.g., cytochrome P450) [52]. Cr (III) ions are unable to transfer back through the plasma membrane, thus increasing intracellular Cr concentrations [53], and free radicals. These free radicals can cause damage to both DNA and intracellular organelles such as mitochondrial, which leads to cell death or mutagenesis. Whilst Cr (III) can directly interact with DNA, Co (II) ions are able to enter the cell via a divalent metal transporter where they also have the potential to cause damage to DNA. Several types of DNA damage are known to occur including chromosomal aberrations, single strand breaks, and oxidative nucleotide. Cr exposure induces cells to undergo apoptosis, but in vitro studies have shown that cells with mitochondrial damage may continue to replicate even though their DNA is damaged by Cr ions, allowing mutagenesis to occur [54].

Generally, implanted metallic cobalt is classed 2B, ‘possibly carcinogenic to humans’ and the implanted metallic chromium is ‘not classifiable' by the International Agency for Research on Cancer (IARC) [55]. However, hexavalent chromium-containing compounds are class 1 (carcinogenic to humans) [54]. Studies of tissues surrounding MoM implants have demonstrated raised levels of DNA damage such as translocations and aneuploidy [56,57,58]. Epidemiological studies have, however, revealed no direct link between MoM prostheses and cancer [59].

Interestingly, there are few incidences of osteolysis reported for MoM implants compared to UHMWPE-containing implants [60,61]. This may be due to the fact that a majority of wear particles identified in vivo are less than 100 nm in size, and are thus much smaller than the UHMWPE particles [42]. In contrast to the osteolysis in the tissues surrounding UHMWPE containing implants, there are rarely multinucleated giant cells present, and only low levels of TNF-α are observed. Nonetheless, severe osteolysis can occur in the MoM prosthesis in some patients with metal hypersensitivity [62].

Some patients after MoM implants have reported pain in their hips/groin upon weight bearing, in which case cystic masses, largely composed of areas of necrosis, are often found around the implants. This response is typically seen in the context of a delayed hypersensitivity reaction, and suggests that a type IV immune response to the metals in the prostheses plays a role in their pathogenesis [63]. Koper MSC et al. (2016) reviewed 160 large-head Magnum M2 MoM THAs in 150 patients and found that 8.75% of the cases had a pseudotumour formation around the prostheses [64].

Only a small number of patients with MoM implants develop Aseptic Lymphocyte Vasculitis Associated Lesions (ALVAL). These patients typically present with severe pain around the prostheses within 3 years of implantation. However, there was very little wear and/or particles in the implants and/or surrounding tissues, which contained a greater number of T cells, B cells, and plasma cells, although only a few macrophages were present [16,65]. The sensitisation and activation of T lymphocytes by antigen-presenting cells (APCs) result in type IV hypersensitivity reactions [54]. APCs present metal-peptide ion complexes to CD4+ T lymphocytes [66]. Both macrophages and T lymphocytes secrete various cytokines including IL-2, IFN [67], IL-1, TNF-α [68], and RANKL [69]. Hallab NJ et al. (2001) investigated human lymphocyte reactivity to metal-protein complexes and found that the reactivity to Cr from Co-Cr-Mo alloy degradation was approximately 10-fold greater than that to the titanium (Ti)- based implant alloy in the higher molecular weight serum proteins [66].

### 3.3. Alumina Ceramics Wear Particles

The biomaterial ceramic is the hardest biomaterial implant used in THA, which produces minimal wear particles and has little or no toxic effect on the body [70]. It is also highly durable with a wear rate approximately 1000-fold lower than UHMWPE-Metal implants [71], and thus is commonly recommended for using in younger THA patients. During normal wear conditions, particulate debris can range from 5–25 nm; however, damage to components due to regionalized overloading or scuffing can create a greater number of debris, where the size of recovered particles are distributed in a bimodal manner (5–25 nm and 14–70 µm) [72,73]. One major problem associated with the use of this material in THA is the squeaking which occurs during movement. Although these noises can disappear overtime, revision surgery is necessary if the squeaking is intolerable [74].

There has been very limited research done to directly compare the biological response to ceramic wear particles with metal and UHMWPE particles. Alumina wear particles have been shown to be well tolerated both in bulk and particle forms [75,76], in vivo and/or in vitro. There is only limited and short-lived inflammatory response to the alumina wear particles [77,78]. Germain et al. (2003) compared the effects of clinically relevant CoCr and alumina ceramic wear particles on the viability fibroblast and histocytes, and determined whether CoCr wear particles at 0.005–50 µm^3^ per cell reduced the cell viability [79]. The alumina ceramic particle volume at 50 µm^3^ per cell were required to cause a reduction in the cell viability [79]. A foreign body reaction was only observed in cases with large amounts of wear particles [80]. An in vitro study by Tsaousi et al (2010) showed that alumina wear particles were very weakly cytotoxic to primary human fibroblasts when compared to CoCr particles [81]. Osteolysis and implant failure are associated with poor implant design and/or implantation techniques. Petit et al. (2002) demonstrated that alumina wear particles are 8–10 times less likely to induce the release of osteolytic cytokines, such as TNF-α, compared with equivalent polyethylene particles [82]. Ceramic particles induce faster macrophage apoptosis than polyethylene (PE) particles, and therefore it was hypothesised that alumina may have the ability to induce macrophage apoptosis, which explains the lower TNF-α release and the differences seen in osteolysis patterns of ceramic-on-ceramic versus metal-on-PE articulations [82].

Alumina ceramic particles generated under microseparation conditions are capable of inducing osteolytic cytokine production by human mononuclear phagocytes. However, the volumetric concentration of the particles needed to generate this response is extremely high; it is unlikely that this concentration threshold is reached in vivo, even under severe microseparation conditions and considering the low wear rates of alumina ceramic [83].

## 4. Conclusions

In conclusion, the immunological responses to different THA biomaterials in bulk and particulate forms vary substantially. The response to the bulk implant biomaterials is relatively acute and similar between UHMWPE, CoCr, and alumina ceramics. However, the response to different biomaterials’ wear particles differ significantly due to the variations in material biocompatibility, size, and volume. Studies to date indicate that alumina ceramics are the most biocompatible, while UHMWPE and CoCr particles have reduced biocompatibility. Therefore, it is very important, in developing future THA devices, to aim to reduce the production of wear particles and to overcome the biological responses elicited by these biomaterials, which will ultimately result in a longer serving implant, reduced revision surgery rate, and the relief of the current socioeconomic impact in our healthcare systems.
